# Generalized Morphea Coincident With Aplastic Anemia: A Case Report

**DOI:** 10.7759/cureus.20955

**Published:** 2022-01-05

**Authors:** Madhusudan R Tapdia, TT Favas, Vijaya Nath Mishra, Abhishek Pathak, Varun K Singh

**Affiliations:** 1 Department of Neurology, Institute of Medical Sciences, Banaras Hindu University, Varanasi, IND

**Keywords:** autoimmunity, generalized morphea, localized scleroderma, aplastic anemia, morphea

## Abstract

Morphea is a rare skin condition characterized by erythematous or violaceous lesions as well as sclerotic plaques. Patients with morphea frequently have other autoimmune disorders. Contributing factors are thought to be autoimmunity and an increase in extracellular matrix production. A case of a 45-year-old male patient with progressive restriction of both shoulder movements and patchy discoloration over the abdomen, neck, back, forearms, and bilateral axillae is discussed in this article. Examination revealed multiple shiny hyperpigmented to hypopigmented indurated plaques, and some lesions showed erythematous to violaceous borders, fine scales, and woody induration. The neurological examination was normal. Skin biopsy showed a sparse superficial perivascular lymphohistiocytic infiltrate with thickening of collagen bundles that were hyalinized in the reticular dermis, which was consistent with superficial morphea. Hematological tests showed pancytopenia and bone marrow aspiration revealed hypocellular marrow, which was consistent with aplastic anemia. The patient was diagnosed with generalized morphea with aplastic anemia. The patient was referred to a transplant center for further treatment, but, unfortunately, he died of sepsis while waiting for his transplant. Our case may indicate a possible link between aplastic anemia and generalized morphea. Due to a possible similar underlying mechanism of pathogenesis, treatment of aplastic anemia may be effective in morphea also. Aplastic anemia must be detected early to reduce complications and mortality in patients.

## Introduction

Morphea is an uncommon skin condition characterized by erythematous or violaceous lesions and sclerotic plaques [1]. It is also known as localized scleroderma but is not associated with systemic involvement like systemic scleroderma [2]. Autoimmunity and increased extracellular matrix production are considered to be implicated in the etiology, but the specific mechanism is uncertain. Patients with morphea have a high incidence of concomitant autoimmune disorders [3,4]. Aplastic anemia is an immune-mediated disorder that is characterized by pancytopenia and severe bone marrow failure [5]. Eosinophilic fasciitis is reported in some aplastic anemia patients, and it is considered a part of the severe manifestation of morphea [6]. Here, we discuss a case of a 45-year-old male patient who presented with generalized morphea and was diagnosed with aplastic anemia after further assessment.

## Case presentation

A 45-year-old male with no prior comorbidities was admitted to the neurology ward with a one-year history of progressive restriction of both shoulder movements. The patient also had patchy discoloration, first noticed over his lower abdomen 14 months back, and over the next two to three months, similar skin changes appeared on the neck, back, forearms, and bilateral axillae. There was no history of pruritus or pain around the lesion. There was no history of dyspnoea, pain, or discoloration of the fingers and/or the toes when exposed to cold. His family, social, and occupational histories were irrelevant.

**Figure 1:**
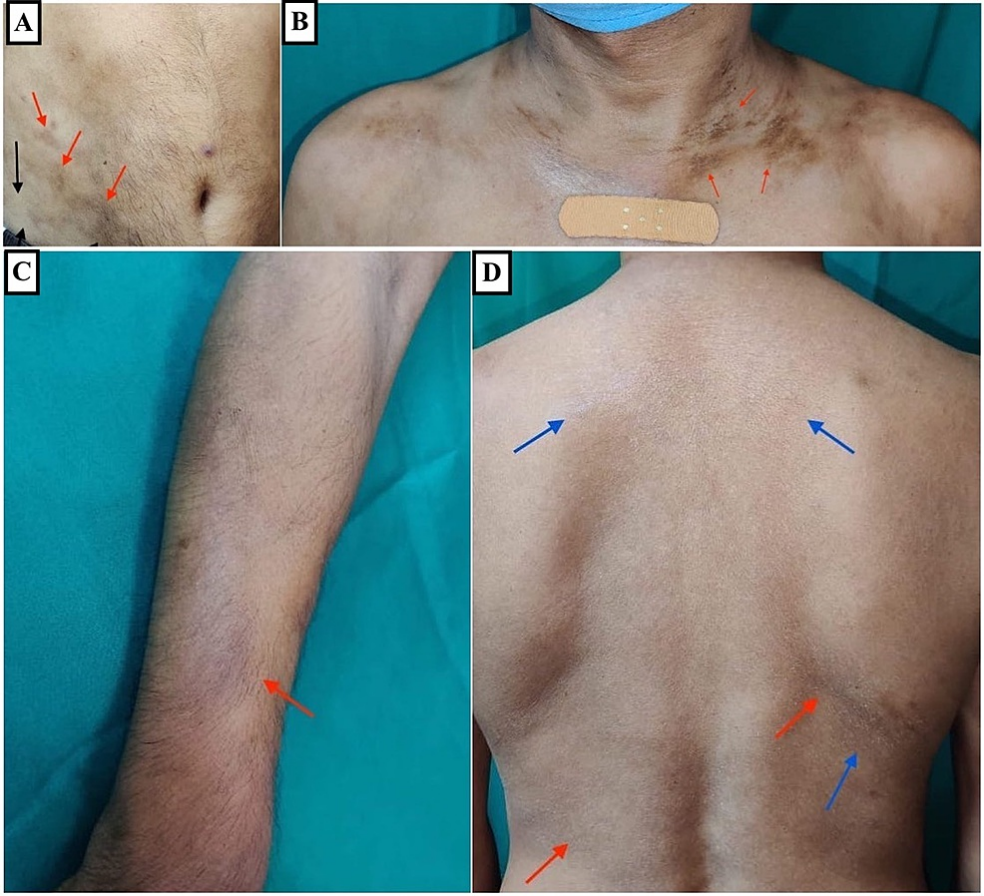
Multiple hyperpigmented (red arrows) to hypopigmented (black arrows) indurated plaques over (A) the lower abdomen, (B) upper chest and neck, and (C) right forearm. (D) Fine scaly lesions over the back (blue arrows).

**Figure 2:**
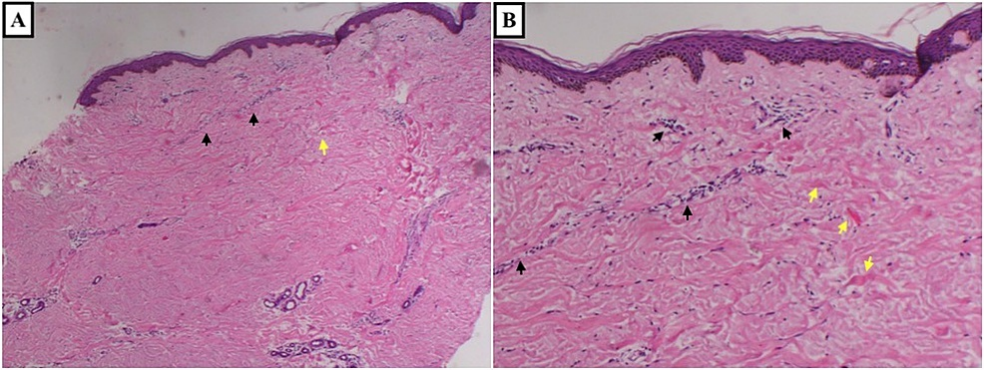
Skin punch biopsy stained with hematoxylin and eosin, showing superficial perivascular lymphohistiocytic infiltrate (black arrows) and thickened hyalinized collagen bundles in reticular dermis (yellow arrows) consistent with morphea. (A) At 4x magnification. (B) At 10x magnification.

General examination revealed pallor. Multiple shiny hyperpigmented to hypopigmented indurated plaques ranging in size from 2 x 2 cm to 15 x 3cm were found over the lower abdomen, upper chest, neck, forearms, back, and bilateral axillae. Erythematous to violaceous borders and fine scales were appreciable over some lesions of the lower abdomen and back, respectively (Figure 1). On the neck, face, and shoulders, there was woody induration over the skin lesions. On neurological evaluation, the patient had Medical Research Council (MRC) grade 5 power over all the joints. Deep tendon reflexes were normal. The examinations of the abdominal and respiratory systems were also normal. A differential diagnosis of generalized morphea and scleredema of Buschke was made and to establish the diagnosis, three deep punch biopsy samples were collected from the most indurated parts of the lesion (right lower abdomen, posterior axillary, and paraspinal skin), which included both hypopigmented and hyperpigmented lesions. All three biopsies showed sparse superficial perivascular lymphohistiocytic infiltrate with thickening of collagen bundles that were hyalinized in the reticular dermis, features consistent with superficial morphea. There was no septal thickening or deep lymphoplasmacytic infiltrate (Figure 2).

Hematological tests showed pancytopenia, normal renal and liver function tests, and lactate dehydrogenase levels. The coagulation panel and viral panels including hepatitis B, hepatitis C, HIV, cytomegalovirus, parvovirus B19, Venereal Disease Research Laboratory (VDRL), and rapid plasma reagin (RPR) were all negative. Anti-nuclear antibody (1:160) was positive, while antibodies against double-stranded DNA, Smith, Scl-70, Sjögren's syndrome-related antigen A/Sjögren's syndrome-related antigen B (SSA/SSB), and ribonucleoprotein (RNP) were all negative. Bone marrow aspiration showed hypocellular marrow consistent with aplastic anemia. The patient was diagnosed with generalized morphea with aplastic anemia based on clinical characteristics, biopsy, and bone marrow findings. The patient was referred to a transplant center for further management. Unfortunately, the patient expired due to sepsis while awaiting his transplant.

## Discussion

Morphea is an idiopathic sclerotic skin disease with heterogeneous phenotypes. It is also known as localized scleroderma [1]. Laxer and Zulian [2] classified it into five subtypes: circumscribed morphea, linear morphea, generalized morphea, pansclerotic morphea, and mixed morphea. Four or more larger than 3 cm lesions that involve at least two out of seven anatomic sites are classified as generalized morphea [1]. The likely pathogenesis in morphea is autoimmunity, and the high incidence of concomitant autoimmune diseases supports this [3,4]. The strongest association is found between generalized morphea and, to a lesser extent, mixed types. Autoimmune disorders reported in morphea are inflammatory bowel disease, type 1 diabetes mellitus, psoriasis, vitiligo, alopecia areata, multiple sclerosis, rheumatic diseases, systemic lupus erythematosus, autoimmune thyroiditis, Meniere's disease, celiac disease, anti-phospholipid syndrome, Still's disease, mixed connective tissue disease, spondyloarthropathy, rheumatoid arthritis, Sjogren's syndrome, morphea, and scleroderma [3]. Morphea is associated with the presence of specific human leukocyte antigen (HLA) class II allele DRB1*04:04 and class I allele HLA-B*37 [7]. Hence, morphea patients are considered for comprehensive workup of concomitant autoimmune syndromes and systemic immunosuppressive agents [3].

Acquired aplastic anemia is a rare immune-mediated hematopoietic disorder associated with life-threatening bone marrow failure. It usually presents with fatigue, shortness of breath, recurrent infections, and bleeding manifestations secondary to pancytopenia. It is characterized by hypocellular bone marrow [5]. A rare association with diffuse eosinophilic fasciitis (Shulman disease), celiac disease, and systemic scleroderma has been reported [6,8,9]. Eosinophilic fasciitis is considered a part of the morphea spectrum [1]. The association of aplastic anemia with generalized morphea is rare and only one previous case has been described, to the best of our knowledge [10]. Another case reported a pansclerotic morphea with red cell aplasia and immune-mediated thrombocytopenia, which improved with antithymocyte globulin [11].

Our patient presented with generalized morphea, without any typical symptoms of pancytopenia, and on evaluation was detected to have aplastic anemia. This case may indicate a possible association between aplastic anemia and generalized morphea. More research is needed to confirm that both diseases share an underlying mechanism that causes sclerosis in the body, which affects both the blood and the skin.

Another important aspect of this association is identifying the appropriate treatment. Antithymocyte globulin, an immunosuppressive therapy traditionally used to treat aplastic anemia, has been shown to successfully treat pansclerotic morphea [11]. Furthermore, recent evidence has confirmed widespread fibrosis responding to immunosuppressive therapy and bone marrow transplantation [11,12]. The position statement released in 2018 by the American Society for Blood and Marrow Transplantation cites evidence finding marked improvement in both cutaneous symptoms and solid organ fibrosis in trial patients after receiving a bone marrow transplant, compared to non-transplanted controls [12]. Unfortunately, our patient could not undergo a transplant and sustained death while awaiting treatment. A successful treatment, on the other hand, may have revealed the common pathophysiology of the disease.

## Conclusions

Physicians should be aware of this association since early identification of aplastic anemia is critical in preventing complications and mortality in patients. Also, hematologic workup should be done in patients presenting with morphea or other sclerotic skin diseases. Aplastic anemia can be managed with bone marrow transplantation or other immunosuppressive therapies, and morphea may also respond to the same treatment due to a possible similar underlying mechanism of pathogenesis.
